# RNA sequencing and bioinformatics analysis of blood from patients with cortical cataracts

**DOI:** 10.1186/s12886-026-04764-2

**Published:** 2026-03-26

**Authors:** Qian Li, Lili Zhu

**Affiliations:** 1 Department of Ophthalmology, The First People’s Hospital of Yuhang District, Hangzhou, China; 2 Department of Ophthalmology, The First Affiliated Hospital, Zhejiang University School of Medicine, Hangzhou, 310000 China

**Keywords:** Cortical cataract, RNA-seq, GSEA, MPO

## Abstract

The cortical cataract is the primary type of age-related cataract and can cause severe vision impairment in the early stage. To explore potential blood markers associated with individual physical conditions in cataract patients, we performed RNA sequencing to compare the differentially expressed genes (DEGs) between cortical and refractive cataract patients. Gene Ontology (GO) enrichment analysis, Kyoto Encyclopedia of Genes and Genomes (KEGG) pathway enrichment analysis, and gene set enrichment analysis (GSEA) were conducted. ELISA was subsequently used to validate differences in the protein expression levels of the selected genes. A total of 311 upregulated and 268 downregulated genes were identified. After bioinformatics analysis, we identified three key DEGs (MPO, CXCL8, and FN1) and determined their possible molecular mechanisms. As determined by ELISA, the expression of these genes increased; among them, MPO was the most significant DEG, and it related to oxidative stress responses through peroxidase activity and the catabolism of hydrogen peroxide. In conclusion, MPO, CXCL8 and FN1 were regarded as potential blood markers in patients. However, owing to the small sample size, the above conclusions are hypothetical.

**Supplementary Information:**

The online version contains supplementary material available at 10.1186/s12886-026-04764-2.

## Introduction

Age-related cataracts (ARCs) are a multifactorial disease and are regarded as the primary cause of blindness worldwide, with a reported prevalence of 17.2% [1]. Individuals with visually significant cataracts tend to experience symptoms such as blurred vision, loss of contrast, halos, and difficulty with glare, which can considerably affect the ability of patients to perform day-to-day tasks [2]. As the primary type of ARC, the age-related cortical cataract (ARCC) is characterized by vacuole formation, which is observed in the anterior or equatorial cortical regions of the lens under slit-lamp microscopy and often leads to visual impairment in the early stage [2, 3]. The incidence of ARCCs is elevated in middle-aged individuals without chronic illnesses or risk factors; however, until recently, why some individuals who are otherwise healthy have an increased risk of developing cataracts before the age of 60 has been unclear. Studies have reported that ARCCs are influenced by multiple risk factors, including ageing, ultraviolet B radiation exposure, and lifestyle factors. When cells are exposed to these conditions, the initiation of oxidative stress causes epithelial and fibre cell apoptosis, ferroptosis and autophagy, all of which negatively affect lens transparency [4, 5]. Concurrently, systemic inflammation exacerbates oxidative injury. Chronic inflammatory states, often linked to conditions such as diabetes, hypertension, and obesity, increase circulating levels of proinflammatory cytokines (e.g., IL-6 and TNF-α) and biomarkers such as the systemic immune-inflammation index (SII), neutrophil-to-lymphocyte ratio (NLR), and platelet-to-lymphocyte ratio (PLR) [6, 7] These factors perpetuate a cycle of oxidative damage and protein misfolding, partly through endoplasmic reticulum (ER) stress pathways and unfolded protein response (UPR) activation, which are also implicated in neurodegenerative and metabolic disorders [8, 9]. Epidemiological evidence further supports the lens as a biomarker of systemic health. For example, metabolic syndrome components—especially hypertension and diabetes—are independently associated with cortical and posterior subcapsular cataracts [10]. A study of older Australian women revealed that systemic conditions such as hypertension and skin cancer, rather than traditional lifestyle factors, were linked to ARCs among very elderly people, highlighting age-specific risk patterns [11]. Moreover, the “LensAge” concept, proposed by Li et al. [6], quantifies biological ageing through lens transparency and is correlated with mortality and age-related diseases, suggesting that the lens reflects cumulative systemic stress. On the basis of this research, we hypothesized that the degree of lens transparency might be related to individual conditions and aimed to identify blood-based biomarkers associated with early-onset cataracts to predict and intervene in their development.

With the development of RNA sequencing (RNA-seq), the transcriptome is often used to measure the total number and type of changes in transcript expression in various diseases [12]. Through bulk RNA sequencing, a comprehensive analysis of organism-wide ageing dynamics revealed that during ageing, gene sets are expressed similarly across tissues and that expression shifts in distinct tissues are strongly correlated with changes in the corresponding protein levels in the plasma [13]. Therefore, we performed RNA sequencing to identify differentially expressed RNAs in blood to explore the function of the differentially expressed genes (DEGs) and the potential pathological processes in ARCC patients.

## Materials and methods

### Human samples

Approval for this research was obtained from the Ethics Committee of The First People’s Hospital of Yuhang District, Hangzhou (Approval Date of the Ethics Committee: January 14, 2025 and Approval No.: 2025–001-06),we recruited patients from February 1, 2025, to April 30. Transcriptome sequencing began immediately after the first cohort of 3 volunteers was enrolled on February 26, 2025, while an additional 10 patients for each group were subsequently recruited to prepare for further Elisa and qPCR.The cohort used for ELISA and qPCR validation was independently recruited and did not overlap with the RNA-seq discovery cohort. ELISA and qPCR experiments were performed blinded to clinical group. Blood samples were taken from patients aged 60 to 70 years after they provided signed informed consent, and all methods were in accordance with the guidelines of the Ethics Committee. A digital slit lamp photographic system was used to acquire digital images of the patients. In accordance with the Lens Opacities Classification System III (LOCS III), a cortical cataract can be divided into stages C1 to C5. C1 represents normal transparent lenses, whereas C3 to C5 indicate severe cataracts [14]. The control group included refractive cataract patients at stage C1, and the ARCC group included patients at stages C3–C5. The exclusion criteria included any retinopathy, high myopia, uveitis, eye trauma, glaucoma, or systemic diseases such as diabetes and autoimmune diseases. Blood samples were obtained by the same experienced nurse before surgery. The supernatant was collected by centrifugation of whole blood and used for both RNA sequencing, ELISA and qPCR. The centrifugation conditions were as follows: centrifugal force/speed: 1500–2000 × g; time: 10–15 minutes; temperature: 4 °C. The cohort used for ELISA and qPCR validation was independently recruited and did not overlap with the RNA-seq discovery cohort. All the images of patient lenses were obtained under a slit-lamp microscope after compound tropicamide eye drops were applied.

### RNA extraction, library construction, and sequencing

Total RNA was extracted using TRIzol reagent (Cat. #15596018; Thermo Fisher Scientific, USA). After RNA extraction, globin RNA was removed using the GLOBINclear kit (or a similar method).The RNA concentration, purity, and integrity were evaluated with an Agilent 2100 Bioanalyzer and an RNA 6000 Nano LabChip kit (Cat. #5067–1511; Agilent, USA). High-quality RNA samples with an RNA Integrity Number (RIN) >7.0 were selected for library construction. Polyadenylated mRNA was enriched from 5 μg of total RNA through two rounds of purification using Dynabeads Oligo(dT) (Thermo Fisher Scientific, USA). The enriched mRNA was fragmented at 94 °C for 5–7 minutes in the presence of divalent cations (Magnesium RNA Fragmentation Module, Cat. #E6150S, NEB, USA). The fragmented RNA was reverse transcribed into cDNA using SuperScript™ II Reverse Transcriptase (Cat. #1896649, Invitrogen, USA). Second-strand cDNA synthesis was then performed with E. coliDNA Polymerase I (Cat. #M0209S, NEB, USA), RNase H (Cat. #M0297S, NEB, USA), and a dUTP Solution (Cat. #R0133, Thermo Fisher Scientific, USA) to generate U-labeled dsDNA. After A-tailing and adapter ligation, size selection was carried out with AMPure XP beads. The U-labeled strand was digested with USER Enzyme (Cat. #M0280S, NEB, USA), followed by PCR amplification to construct the final sequencing libraries, which had an average insert size of 300 ± 50 bp. Finally, paired-end sequencing (2 × 150 bp) was performed on an Illumina NovaSeq 6000 platform (LC-Bio Technology Co., Ltd., Hangzhou, China) according to the manufacturer’s protocol.

### Bioinformatic analysis

Data preprocessing and alignment. Raw sequencing data (FASTQ files) were first subjected to quality assessment using FastQC (v0.12.1). Adapter sequences and low-quality bases (Phred score < 20) were trimmed using Trimmomatic (v0.39). The cleaned high-quality sequencing reads were then aligned to the human reference genome GRCh38/hg38 using the splice-aware aligner HISAT2 (v2.2.1). The average mapping rate across all samples was 92.5% (range: 89.1% − 95.3%), with an average sequencing depth of 40 million paired-end reads per sample. The alignment results (BAM format files) were sorted, deduplicated, and indexed using SAMtools (v1.20). Based on the GENCODE v44 gene annotation file, read counts for each gene were generated using featureCounts (from Subread package v2.0.6). Gene expression levels were normalized by fragments per kilobase per million mapped reads (FPKM) [15]. Differential expression analysis was performed in the R (v4.4.2) environment using the DESeq2 (v1.48.1) package [16]. Differentially expressed genes (DEGs) were defined as those with an adjusted *p*-value (False Discovery Rate, FDR) <0.05 and an absolute log2 fold change (|log2FoldChange|) ≥1 (corresponding to an absolute fold change |FC| ≥2).

### Functional enrichment and network analyses

Gene Ontology (GO) enrichment analysis of the DEGs was performed using the Kolmogorov-Smirnov test (ks.test()function) in R. Kyoto Encyclopedia of Genes and Genomes (KEGG) pathway enrichment analysis was assessed using KOBAS (v3.0) software with the hypergeometric test, and a significance threshold of *p*-value < 0.05 was applied. Gene Set Enrichment Analysis (GSEA) was conducted using the GSEA (v4.1.0) software and the MSigDB (v2023.2) database. The gene expression matrix was rank-ordered using the Signal2Noise normalization method, and enrichment scores and significance *p*-values were calculated with default parameters. The criteria for significance were: |Normalized Enrichment Score (NES)| >1, nominal *p*-value (NOM *p*-value) <0.05, and false discovery rate q-value (FDR q-value) <0.25. In addition, we performed protein–protein interaction (PPI) analysis via STRING software (http://string-db.org/). The PPI network genes were re-evaluated using the cytoHubba plugin in Cytoscape(http://www.cytoscape.org/, version 3.8.2) based on multiple network centrality algorithms (degree centrality, betweenness centrality, and MCC).

EVenn (http://www.ehbio.com/test/Venn/#/) was used to create Venn diagrams.

Venn Diagram Abbreviations and Definitions

PPI DEGs: The DEGs that are significant in the Protein-Protein Interaction network.

DEGs in KEGG: The top 100 DEGs that play a major role in the KEGG pathway analysis.

DEGs in GO: The top 100 DEGs that play a major role in the Gene Ontology (GO) analysis.

Significant DEGs: The DEGs with a *p*-value < 0.05 and an absolute Fold Change.

### Enzyme-linked immunosorbent assay (ELISA)

To verify the differences in the expression of the DEGs, we measured the levels of their encoded proteins via ELISA. We recruited another 10 volunteers.After the blood was collected from the patients, it was centrifuged at 10,000 × g for 15 min. The supernatant was collected and immediately stored at − 80 °C for later use. The protein levels of all the key DEGs were measured by ELISA according to the manufacturer’s instructions. The colour intensity in a colourimetric reaction was measured at 450 nm. The samples were run in triplicate, and the concentrations were calculated on the basis of standard curves.

### Quantitative real-time PCR (qPCR)

Gene expression levels were quantified using quantitative real-time polymerase chain reaction (qPCR). Specific primers and probes were designed for the target genes. The sequences of the primers are listed below, (5’−3’) : MPO : GCTGGGCTTCATCAACATGG; CXCL8 : ATGACTTCCAAGCTGGCCGTGGCT; FN1: CGGTGGCTGTCAGTCAAAG. GAPDH was used as the endogenous reference gene for normalization due to its stable expression across all blood samples in this study. Total RNA was isolated from cellular samples by TRIzol reagent. A two-step reverse transcription–PCR protocol was used for cDNA synthesis and subsequent amplification. Briefly, RNA templates were first reverse transcribed into complementary DNA (cDNA) using a reverse transcriptase enzyme. The synthesized cDNA served as the template for qPCR amplification, which was carried out with gene-specific primers under the following thermal cycling conditions: initial incubation at 50 °C for 2 min, polymerase activation at 95 °C for 10 min (1 cycle), and 40 cycles of denaturation at 95 °C for 15 s and combined annealing/extension at 60 °C for 1 min. To ensure assay validity, nuclease-free double-distilled H₂O was included as a negative control, while a plasmid containing a known target gene sequence served as a positive control. Fluorescence signals emitted during the amplification process were monitored in real time using SDS software (Applied Biosystems), enabling continuous data acquisition and subsequent analysis of the amplification curves.Normalization of sample variations using internal reference genes,the correction value is calculated as the ratio of the target gene to the internal reference gene, i.e.,**X**^△ - ** Ct**^ , Here, **2**^△ - ** Ct**^ represents the normalized quantity of the target gene molecules in each sample after correction.To compare the expression level of a gene across different samples, the relative value is derived as the ratio of the treated sample to the control sample:Fold Change=**2**^△ - ** Ct1**^**/2**^△ - ** Ct2**^**=2**^△ △**- Ct**^.

### Statistical analysis

The data conformed to a normal distribution as determined by the Shapiro-Wilk test.Two-tailed Student’s t tests were used for statistical analysis. All the data are expressed as the means ± SE, and a P value < 0.05 was considered to indicate statistical significance.

## Results

In all figure labels, “Age” denotes patients with age-related cortical cataract.

### Clinical information of the patients

**Fig. 1:**
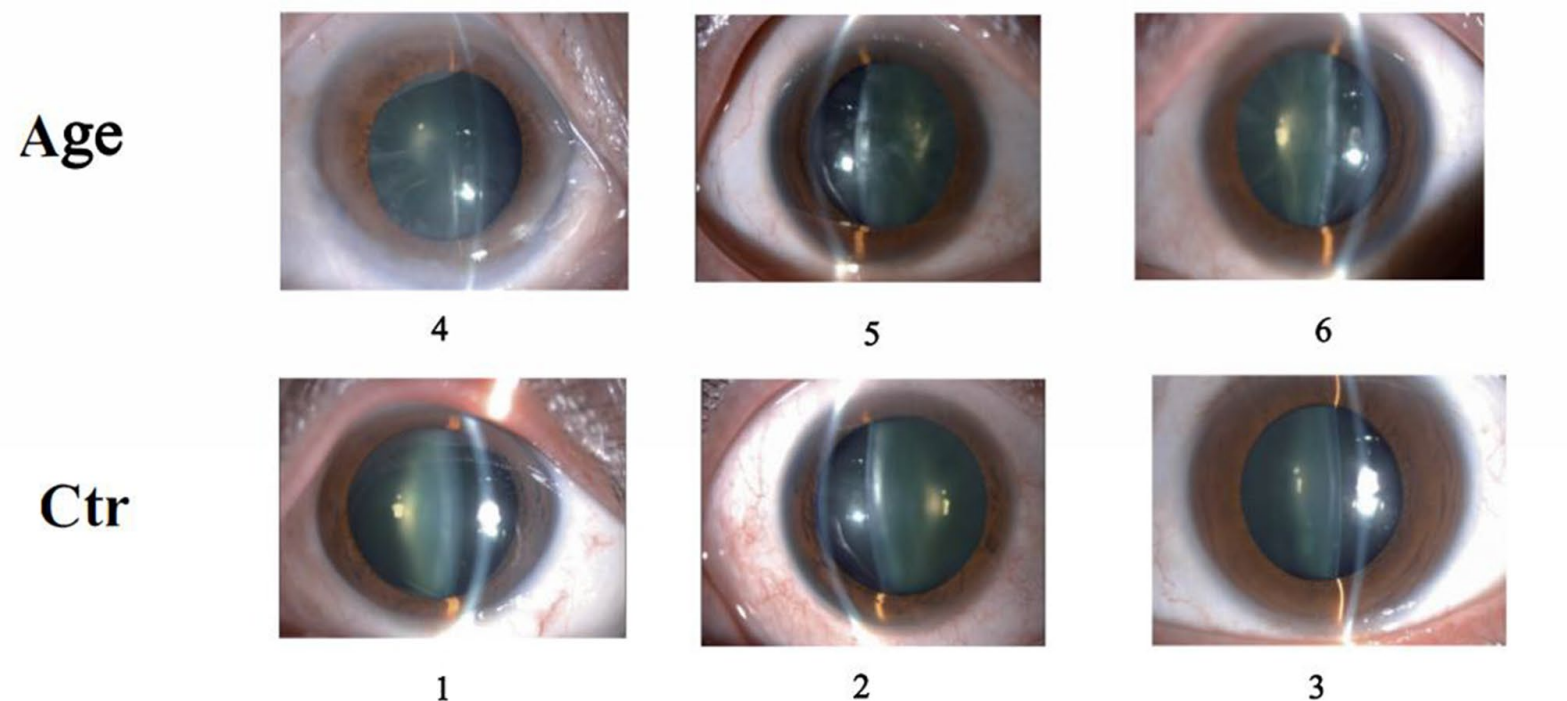
The photo of lens in patients under slitlamp microscopy. The opacity of lenses in ARCC patients are obvious severe than that of the control group

We performed high-throughput RNA sequencing (RNA-seq) on three ARCC patients and three refractive cataract patients, with 10 samples collected from each group for ELISA. Patient information is presented in Table 1. The digital images of patients with different lens opacities clearly differed between the groups (Fig. 1).

**Table 1:** The clinical information of patients

Patient	1	2	3	4	5	6
Eye	Left	Right	Left	Left	Right	Right
Lens	C1	C1	C1	C4	C4	C4

### Analysis of differentially expressed genes (DEGs)

**Fig. 2:**
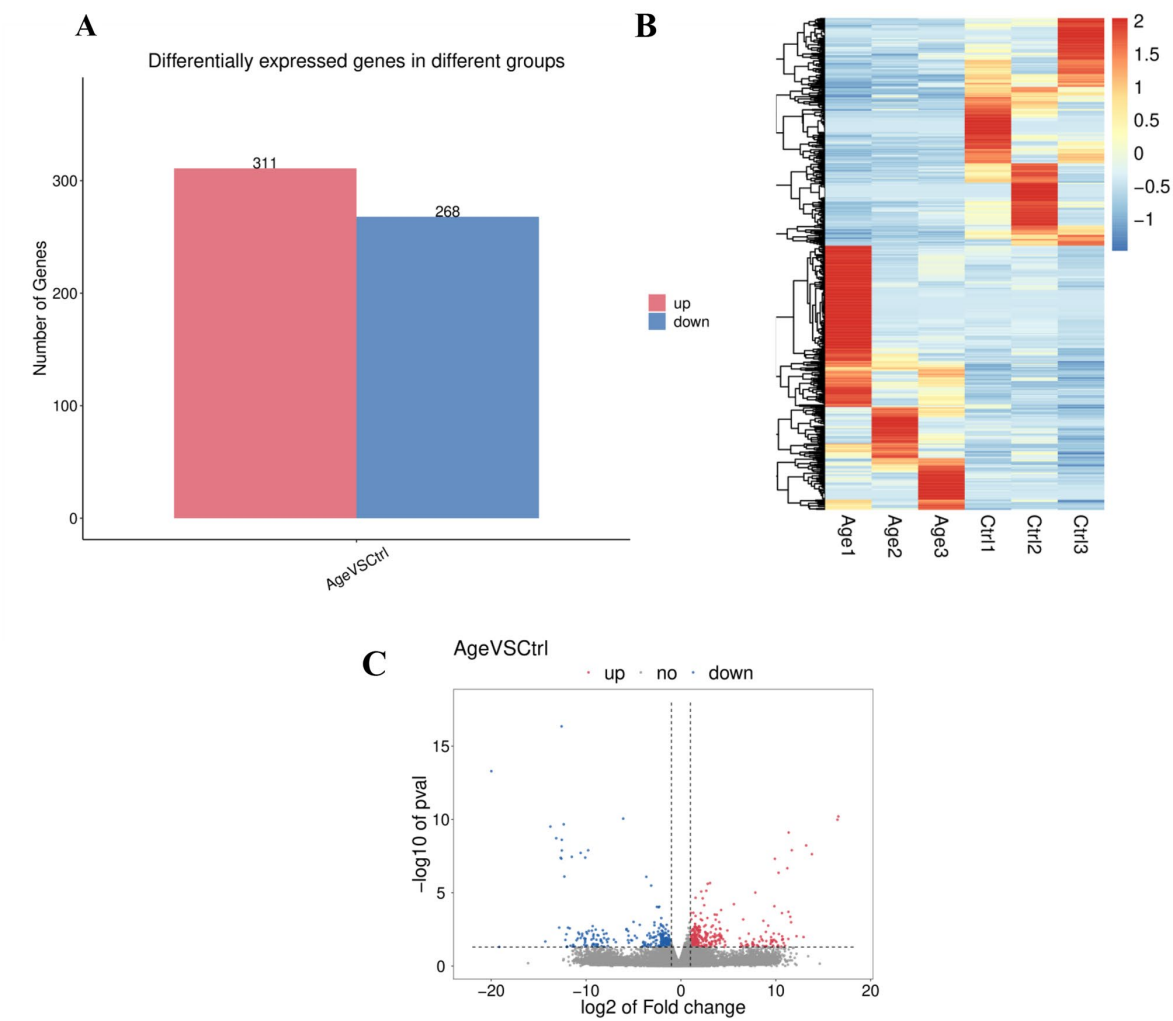
Volcano plot and heatmap of differentially expressed gens between groups. The histogram of DEGs (**A**), the abscissa is the number of genes, the red column presents increased number and the blue column presents decreased number. In heatmap (**B**), each row is a gene, each column is a sample/repeat, and different color represents different quantity of expression. Volcano plot (**C**), the abscissa is the FC in protein concentration and the ordinate is the statistical significance. Blue dots presents upregulated genes and red dots presents downregulated genes; black dots are proteins without significant change

This analysis revealed a total of 579 DEGs between patients and controls, among which 311 were upregulated and 268 were downregulated (Fig. 2A). To better visualize the differential expression of these genes, we generated a volcano plot and heatmap based on the top 100 DEGs. The results revealed that the gene expression levels differed between the groups (Fig. 2B and C).

### Functional enrichment analyses of the DEGs

**Fig. 3:**
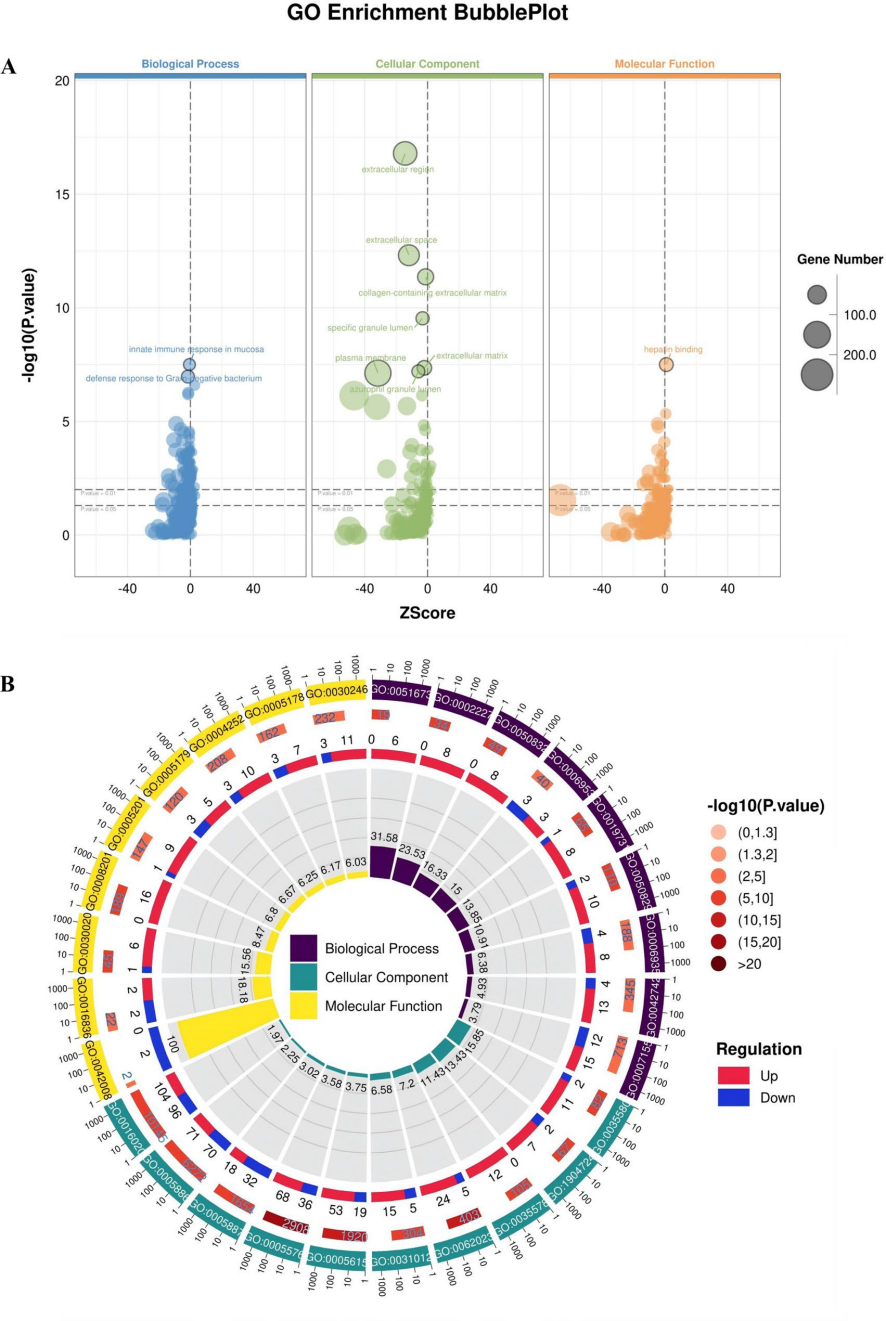

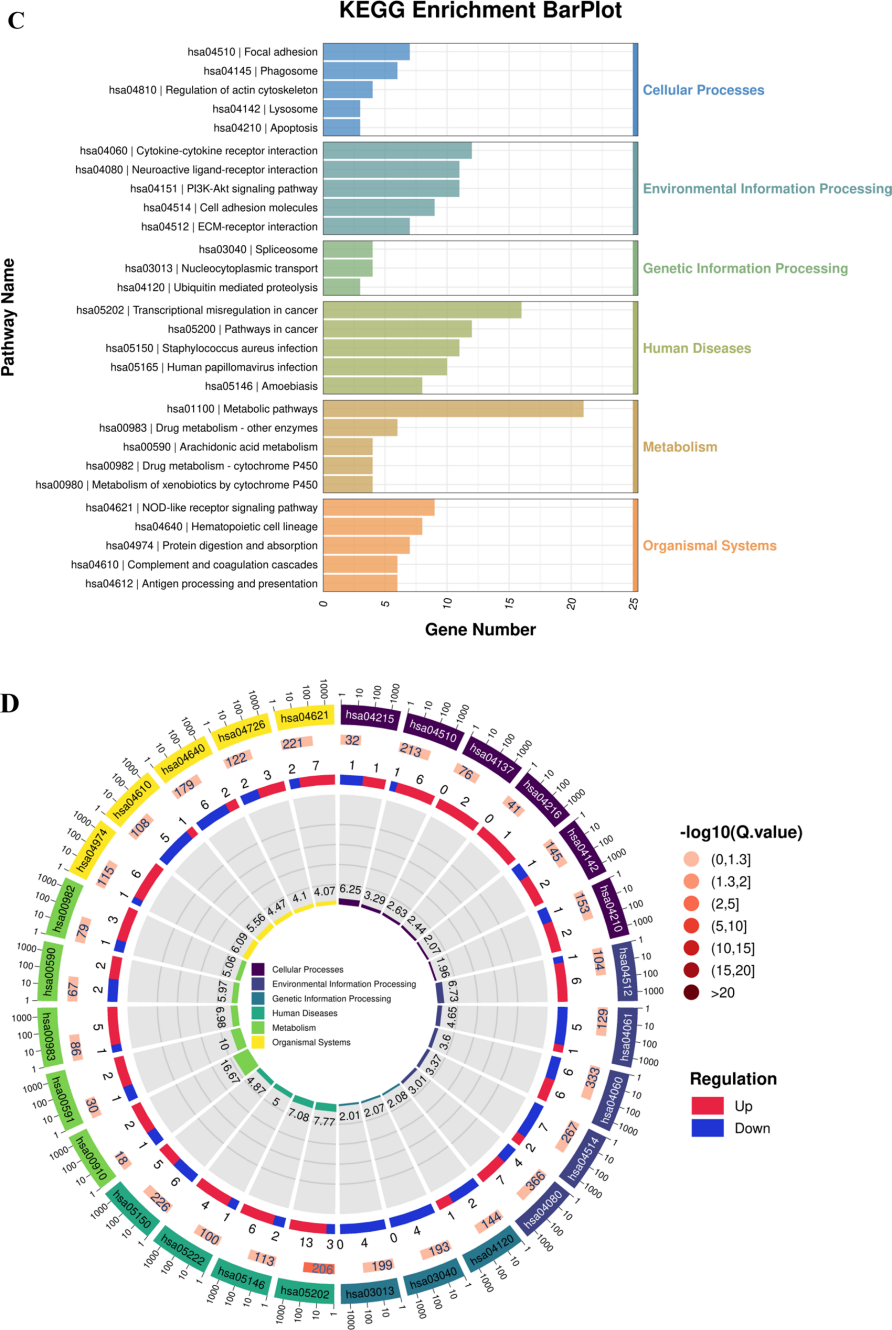
GO and KEGG analysis of DEGs. (**A** bubble plots of DEGs in GO analysis. (**B**-**D**) circular network plots of enriched items in GO and KEGG analysis. (**C**) Bar chart of enriched items for DEGs in KEGG analysis. The selection criteria for GO and KEGG enrichment terms: *p*-value < 0.05

To understand the functions of the DEGs, we performed a GO functional enrichment analysis based on three categories, namely, BP, MF and CC. Bubble plots show the major GO terms in each category (Fig. 3A). Circular network plots show the numbers of DEGs, P values and fold changes in related GO terms (Fig. 3B). The most enriched cellular component term was specific granule lumen (GO:0035580; Rich.Factor = 0.1585; up: *n* = 11; down: *n* = 2); the most enriched biological process term was disruption of plasma membrane integrity in another organism (GO:0051673; Rich.Factor = 0.3158; up: *n* = 6; down: *n* = 0); and the most enriched molecular function term was interleukin-18 receptor activity (GO:0042008; Rich.Factor = 0.2341; up: *n* = 0; down: *n* = 2). In addition, the KEGG enrichment bar plot (based on the gene numbers) and circular network plot (based on rich factors) of the DEGs in the control group are shown in Fig 3C and D. The most enriched cellular process pathway was apoptosis-multiple species (hsa04215; Rich.Factor = 0.0625; main DEGs (up: *n* = 1; down: *n* = 1)); the most enriched environmental information processing pathway was ECM-receptor interaction (hsa04512; Rich.Factor = 0.0673; up: *n* = 6; down: *n* = 1); the most enriched genetic information processing pathway was ubiquitin-mediated proteolysis (hsa04120; Rich.Factor = 0.0208; up: *n* = 1; down: *n* = 2); the most enriched human disease pathway was transcriptional misregulation in cancer (hsa05202; Rich.Factor = 0.0777; up: *n* = 13; down: *n* = 3); the most enriched metabolism pathway was nitrogen metabolism (hsa00910; Rich.Factor = 0.1667; up: *n* = 2; down: *n* = 1); and the most enriched organismal system pathway was protein digestion and absorption (hsa04974; Rich.Factor = 0.0609; up: *n* = 6; down: *n* = 1).

### GSEA of the DEGs

**Fig. 4:**
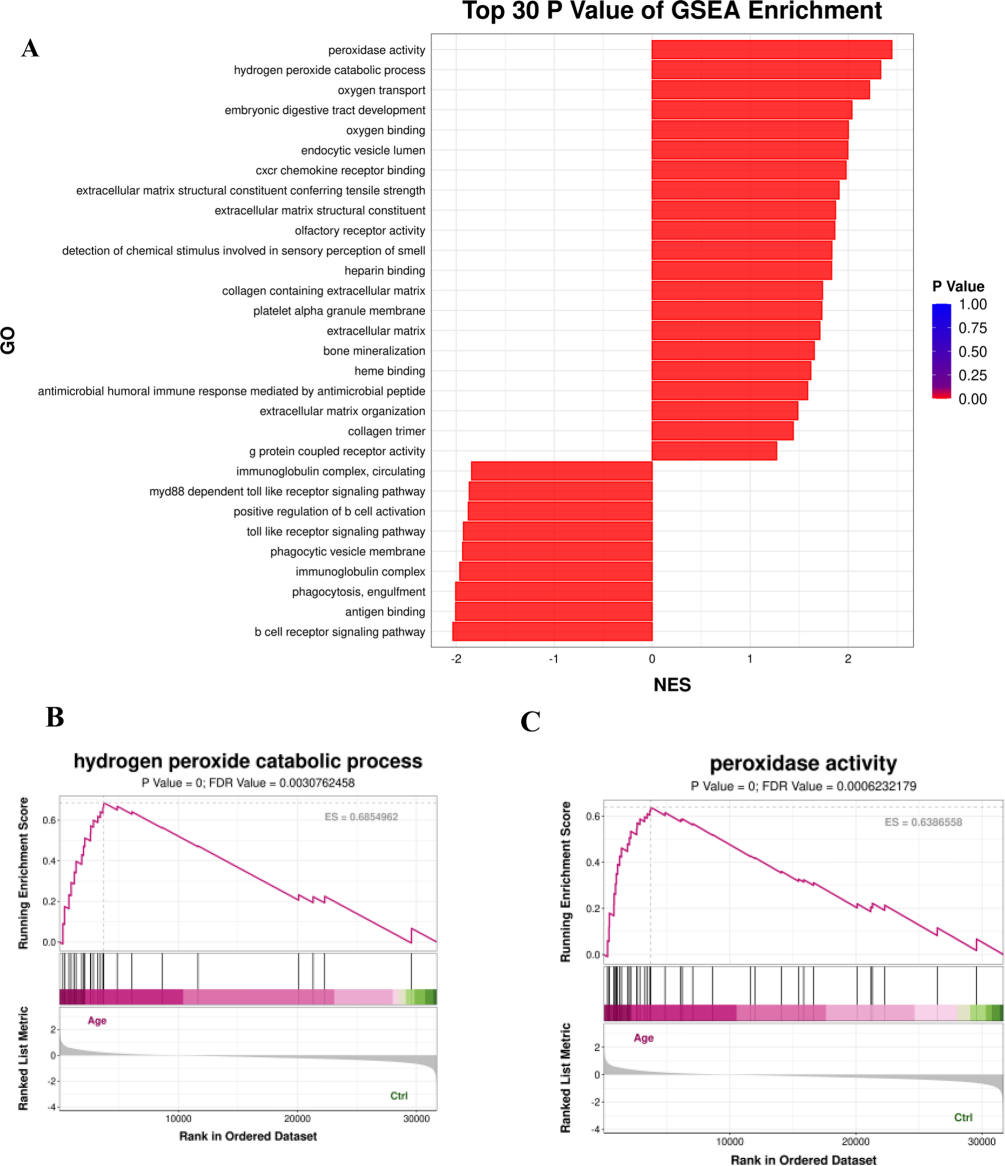
(**A**) Gene set enrichment analysis (GSEA) revealing the top 30 P value terms. (**B**) GSEA revealing the upregulation of peroxidase activity pathways in ARCC. (**C**) the upregulation of hydrogen peroxide catabolic process pathways in ARCC

To gain insight into the changes and functions of the DEGs, we performed GSEA on the basis of the GO analysis results. The top 30 enriched genes according to the gene enrichment analysis (P value) are shown in Fig. 4A, and the top 2 enriched pathways were peroxidase activity and the hydrogen peroxide catabolic process (Fig. 4B and C). Furthermore, we detected significant upregulation of genes (HBD, HBM, and MPO) involved in peroxidase activity and in the catabolic process of hydrogen peroxide, with the exact information shown in Table 2.

**Table 2:** The DEGs in peroxidase activity and hydrogen peroxide catabolic process

Gene_Name	Log2.fc.	*P*-value	Regulation	Significant
HBD	1.362121812	0.028782963	up	yes
HBM	1.532558335	0.003652682	up	yes
MPO	3.564867192	0.010922348	up	yes

### PPI and Venn analyses

**Fig. 5:**
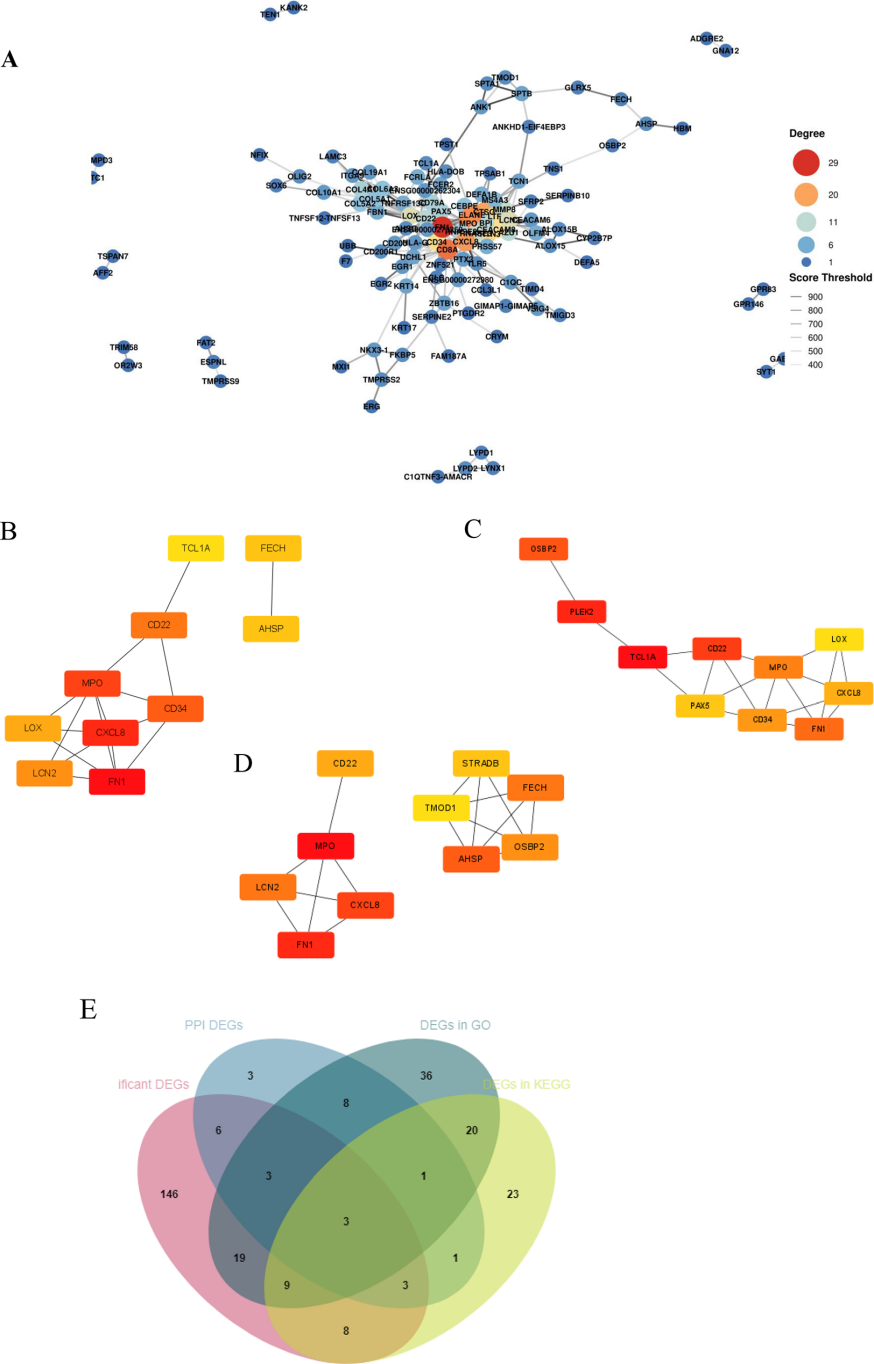
(**A**) the PPI network for DEGs. (**B**):the top DEGs in degree centrality. (**C**):the top DEGs inbetweenness centrality. (**D**):the top DEGs in MCC analysis. The line represents the protein interaction recorded or predicted by STRING, each box represents the key proteins recorded by CytoScape. (**E**)the venn diagram of the DEGs between groups

To determine the molecular mechanism involved in ARCCs, we used STRING software to analyse the DEGs between the groups and then imported the results into Cytoscape software (http://www.cytoscape.org/, version 3.8.2) to further analyse the functional PPI networks. The key PPI network for the DEGs between ARCC and refractive cataract patients is shown in Fig. 5A the results of multiple network centrality algorithms (degree centrality, betweenness centrality, and MCC) were shown in Fig. 5B, C and D. MPO, CXCL8, and FN1 consistently ranked within the top 10 across multiple centrality metrics. Furthermore, we generated Venn diagrams to identify the key proteins (Fig. 5E). Present the specific genetic information in the form of a table (Table 3).

**Table 3:** The results of venn

Gene Name	Change	Significant	log2(fc)	*P* -value	FDR
MPO	up	yes	3.56	0.000000	0.00007
FN1	up	yes	1.90	0.001882	0.01796
CXCL8	up	yes	3.23	0.002107	0.02837

### Validation of the expression of key genes in ARCCs through ELISA and qPCR

**Fig. 6:**
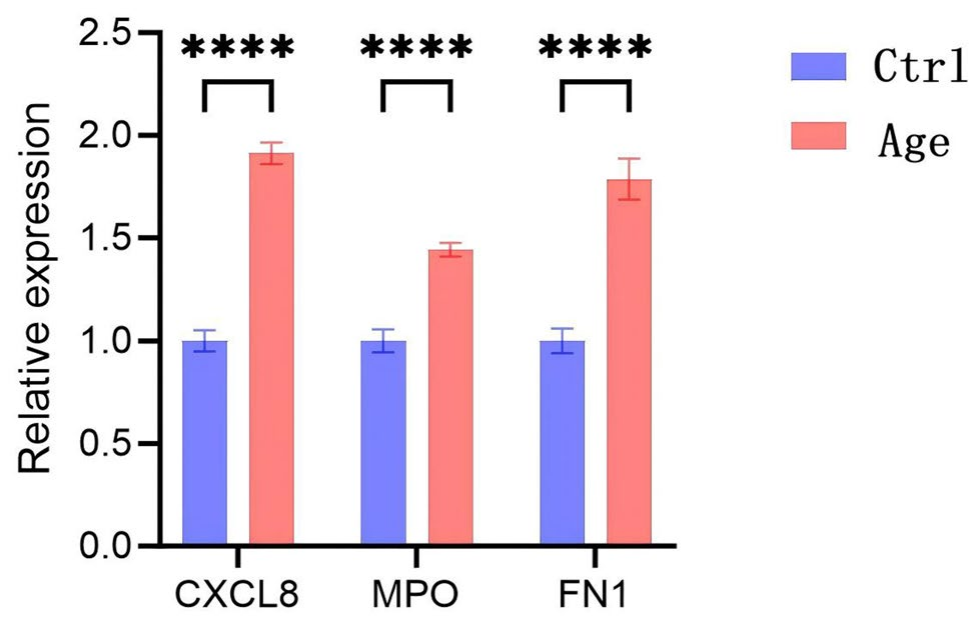
Expression of candidate differential genes was verified by qPCR. The blue bars represent the control group, the red bars represent the ARCC group, the horizontal axis shows the names of different genes, and the vertical axis indicates the relative expression level, ****p < . 0001

**Fig. 7:**
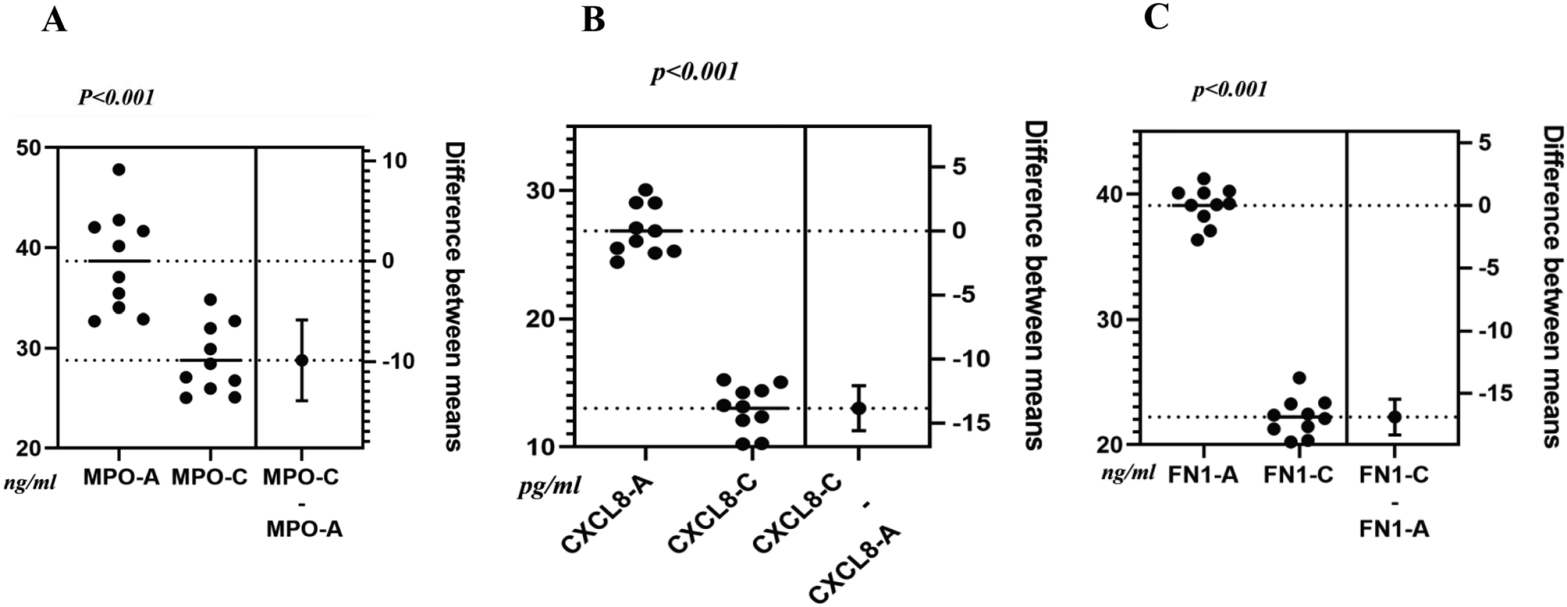
Comparison of candidate genes concentrations and the mean difference between groups. The scatter plot on the left displays individual gene concentration measurements (in ng/ml) for different groups, with the horizontal lines representing the mean concentration for each group. The right panel illustrates the calculated difference between the means of the two groups (gene C minus gene A), plotted along with its confidence interval (or standard error)

After all the bioinformatics analyses were performed, we obtained three key genes, namely, MPO, FN1 and CXCL8, whose expression was upregulated. To further validate our findings, we performed ELISA and qPCR to assess both the RNA and protein expression levels of the target genes. The results demonstrated that the expression of MPO, CXCL8, and FN1 was significantly greater in the cataract group than in the control group, with all p values being less than 0.05. The RNA expression data are presented in Fig. 6, and the ELISA results are shown in Fig. 7.

## Discussion

The primary limitation of this study is the small sample size used for the initial RNA-seq screening, which may affect the stability and generalizability of the differentially expressed genes identified. Therefore, all conclusions should be interpreted as hypothesis-generating and require validation in larger independent cohorts.

The opacity of the lens may differ across patients of the same age, which we hypothesized was influenced by the physical condition of the individual. We compared the expression levels of DEGs in the blood between ARCC and age-matched refractive cataract patients by RNA sequencing (RNA-seq) and ultimately identified 311 upregulated DEGs and 268 downregulated DEGs between the groups. After bioinformatic analysis and PPI analysis, we screened three key DEGs (MPO, CXCL8, and FN1). They play vital roles in the processes of inflammation, oxidative stress and senescence, and their increase indicates pathological changes in ARCC patients and provides new knowledge for measuring individual physical condition through lens transparency.

Myeloperoxidase (MPO) is a haemoprotein that is expressed in the azurophilic granules of neutrophils and in the lysosomes of monocytes and is often released during the acute phase of inflammation, with its elevation indicating increased inflammatory activity in vulnerable coronary plaques [17]. Retinal function is impaired 24 h after infection, and the number of viable bacteria and MPO activity in the eye increase 48 h later [18]. Our study revealed that MPO levels were significantly elevated in the ARCC group, suggesting that MPO may aid in the differential diagnosis between the ARCC and control groups. KEGG analysis and GSEA revealed that MPO was among the key DEGs and was associated with primary KEGG pathways and GSEA terms, indicating that MPO may participate in the pathogenesis and progression of ARCCs. The results of GSEA suggested that MPO expression could lead to oxidative stress reactions through peroxidase activity and the catabolic process of hydrogen peroxide.Many studies have demonstrated that the pathogenesis of cataracts involves oxidative stress and that the lens is equipped with an efficient antioxidant system [19, 20]. The lens is continuously subjected to oxidative stress from both endogenous and exogenous sources. Prolonged exposure to these biological hazards leads to lens opacity and subsequent loss of its high refractive index [21, 22]. Lu et, al [23] study found that under oxidative stress conditions, EGR2 expression was significantly upregulated in lens epithelial cells (LECs). Functional experiments demonstrated that overexpression of EGR2 exacerbated oxidative damage and apoptosis in LECs, while in vivo knockdown of EGR2 using adeno-associated virus (AAV) markedly alleviated lens opacity in a rat model. These studies collectively demonstrate that oxidative stress plays a significant role in the pathogenesis of cataracts. Elevated systemic MPO levels are associated with increased oxidative stress, as MPO generates reactive oxygen species (ROS) and hypochlorous acid (HOCl), relating to protein modification and tissue damage [24]. MPO-driven oxidation products have a long half-life and the ability to diffuse across tissues; therefore, they may accelerate crystalline protein aggregation, liquefaction, and cortical vacuole formation and enable systemic elevation of MPO to directly affect the lens microenvironment [22]. Enzymatic antioxidants within the lens, including superoxide dismutase (SOD), catalase, and glutathione peroxidase (GPx), play critical roles in preventing cataract formation [22, 25]. Systemically elevated MPO or its products may enter the intraocular space by compromising the integrity of the blood-aqueous barrier. Locally within the lens, they can exacerbate oxidative damage to the lens through direct oxidative attacks on the membrane proteins and cytoskeletal proteins (such as crystallins) of lens epithelial cells (LECs), as well as by depleting intracellular antioxidants (such as glutathione). Moreover, systemic conditions such as cardiovascular disease and diabetes are linked to high MPO levels, which may predispose individuals to earlier or more severe lens opacification, independent of age [26, 27]. In summary, our study revealed an increase in MPO levels in the blood of patients with ARCCs, and MPO may cause the formation of ARCCs through inflammation and oxidative stress activity. Previous studies have focused mostly on the redox status within the lens [7]; however, this study proposes the concept of “systemic oxidative stress burden,” emphasizing that blood biomarkers such as MPO reflect the systemic oxidative stress status and that lens changes are merely local manifestations. These findings provide valuable information and insights for risk assessment, early diagnosis, and disease monitoring in ARCC patients.

Chemokine ligand 8 (CXCL8), also known as interleukin-8 (IL-8), is a chemotactic proinflammatory cytokine that is produced by various cell types and is associated with oxidative stress-mediated inflammatory responses in the body [28]. Studies have demonstrated that CXCL8 may induce MCP-1 expression and promote MCP-1 secretion via the phosphatidylinositol 3-kinase (PI3K) and extracellular signal-regulated kinase 1/2 (ERK1/2) pathways [29]. CXCL8 can also be secreted by residual lens epithelial cells, and its upregulation occurs early in posterior capsular opacification through the ERK1/2 pathway in cataract patients [30]. CXCL8 may also increase the expression of matrix metalloproteinases and facilitate epithelial–mesenchymal transition (EMT), contributing to lens fibre disorganization [31]. In addition, the levels of IL-8 increase during age-related diseases, such as tumour metastasis and senescence-associated inflammation [32]. In our study, we revealed that CXCL8 was upregulated in the blood, and PPI analysis indicated that it might play a vital role in the development of ARCCs in patients. These results are consistent with those of previous reports. Therefore, we hypothesize that elevated systemic levels of CXCL8 may recruit and activate circulating neutrophils, promoting their infiltration into the eye. In the lens microenvironment, activated LECs or residual inflammatory cells may also locally produce CXCL8, forming an autocrine/paracrine inflammatory amplification loop. The sustained inflammatory microenvironment further promotes the release of pro-inflammatory factors (such as IL-1β, TNF-α), which can induce epithelial-mesenchymal transition (EMT) in LECs, leading to cell proliferation, migration, and fibrosis. In conclusion, CXCL8 expression is increased in both the aqueous humour and blood of cataract patients, and CXCL8 may participate in inflammation, oxidative stress and senescence during disease development. The correlation between systemic CXCL8 levels and ARCCs reinforces the idea that the lens reflects the overall inflammatory status of the body [33].

Fibronectin 1 (FN1) is a glycoprotein that is found in the extracellular matrix. Research has indicated that FN1 is involved in cellular mechanisms such as cell adhesion, migration, and wound healing promotion and plays a role in enhancing intercellular adhesion to maintain the cytoskeletal architecture [34]. FN1 is a marker of tissue fibrosis and epithelial–mesenchymal transition (EMT) [35, 36]. During the process of EMT, the proliferation and migration of lens epithelial cells increase, leading to the transformation of these cells into fibroblasts, which produce FN1, a key component of the extracellular matrix, ultimately forming opaque plaques beneath the anterior or posterior lens capsule [37]. In cortical cataracts, persistent microinjury or metabolic stress may trigger FN1-mediated pathways, leading to LEC dysfunction and the loss of lens transparency [38]. TGF-β/Smad signalling activation can significantly upregulate the expression of FN1, which is the downstream target gene of the TGF-β/Smad pathway, resulting in abnormal proliferation of posterior subcapsular congenital cataracts (PSCs) in a mouse model [39] Recent experiments have identified and confirmed the central role of the transcription factor ATF6 in maintaining lens epithelial cell homeostasis [40]. This study is the first to demonstrate that ATF6 expression is significantly upregulated in a TGF-β1-induced EMT model. Inhibition of ATF6 with the specific inhibitor Ceapin-A7 effectively reduced the expression of EMT markers (such as FN1, Vimentin, and α-SMA) and cellular fibrosis. In addition, the expression of FN1 is increased in the aqueous humour of proliferative diabetic retinopathy (PDR) patients, and this protein acts as a hub protein according to proteomics analyses [41]. These results suggest that local elevation of FN1 levels is involved in various ocular diseases. In our study, FN1 was upregulated in the blood and was regarded as one of the hub genes. However, few reports exist on the increase in blood levels of FN1 in patients with cataracts or other ocular diseases; thus, how FN1 influences the development of ARCCs is still unknown. Its blood level may indicate the overall trend of tissue repair and fibrotic responses in individuals. In the context of ARCCs, increased blood FN1 may indicate that the lens microenvironment is more prone to EMT-like changes, directly promote the EMT process in LECs by activating downstream signaling pathways such as FAK/PI3K/Akt or TGF-β/Smad via integrin receptors, thereby increasing ARCC susceptibility.

## Limitations

Control group selection: Although refractive cataract patients served as practical controls, comparisons with age-matched healthy controls would strengthen conclusions.

Sample size: Although the data were validated by ELISA and qPCR, the RNA-seq cohort (*n* = 3/group) warrants expansion in future studies.

Mechanistic gaps: Current findings establish associations but require in vitro and in vivo validation of causal relationships.

This experiment selected whole blood rather than aqueous humor or lens epithelial cells for sequencing and validation, as the study was designed to investigate the correlation between systemic alterations and the degree of lens opacity.

## Conclusion

We ultimately identified three key DEGs whose expression was significantly altered in the blood of patients with ARCCs, and these DEGs may accelerate the development of ARCCs. MPO was the most significantly expressed mRNA and may relate to opacity of the lens in ARCC patients through peroxidase activity and the catabolic process of hydrogen peroxide. Increased CXCL8 expression at the local and systemic levels subsequently induces inflammation, oxidative stress, and senescence. Changes in blood FN1 levels are influenced by many factors, and FN1 may be a potential marker in the blood of patients with ARCCs, along with MPO and CXCL8. Biomarkers differ in specificity; traditional biomarkers (e.g., GSH and MDA) mostly reflect the overall level of oxidative stress, whereas MPO, CXCL8, and FN1 possess functional specificity as MPO directly produces oxidants, CXCL8 specifically recruits inflammatory cells, and FN1 directly participates in the fibrotic process. However, these findings are preliminary and derived from computational prioritization; further experimental validation in independent cohorts is needed to confirm their biological and clinical relevance.

## Electronic supplementary material

Below is the link to the electronic supplementary material.

Supplementary material 1

## Data Availability

The datasets generated during and/or analysed during the current study are available from the following. https://www.ncbi.nlm.nih.gov/geo/query/acc.cgi?acc=GSE308087. The Reviewer token: qdcpiaeqrbmbxqh
