# Supplementary material for: RNA sequencing and bioinformatics analysis of blood from patients with cortical cataracts
Source: BMC Ophthalmol. 2026 Mar 26;26:231. doi: 10.1186/s12886-026-04764-2 (PMC13141249; doi:10.1186/s12886-026-04764-2)
Supplement: Supplementary file 1 — Supplementary material 1 [file 12886_2026_4764_MOESM1_ESM.docx]

Supplement 1

| **qPCR Normality test** | | | | | | |
| --- | --- | --- | --- | --- | --- | --- |
|  | Kolmogorov-Smirnov^a^ | | | Shapiro-Wilk | | |
|  | test statistic | df | Sig. | test statistic | df | Sig. |
| MPO.2 | .190 | 10 | .200^*^ | .914 | 10 | .313 |
| MPO.1 | .139 | 10 | .200^*^ | .932 | 10 | .467 |
| CXCL8.1 | .171 | 10 | .200^*^ | .915 | 10 | .318 |
| CXCL8.2 | .152 | 10 | .200^*^ | .919 | 10 | .348 |
| FN1.1 | .205 | 10 | .200^*^ | .942 | 10 | .577 |
| FN1.2 | .138 | 10 | .200^*^ | .950 | 10 | .663 |

Supplement2

| **Elisa Normality test** | | | | | | |
| --- | --- | --- | --- | --- | --- | --- |
|  | Kolmogorov-Smirnov^a^ | | | Shapiro-Wilk | | |
|  | test statistic | df | Sig. | test statistic | df | Sig. |
| MPO.2 | .185 | 10 | .200^*^ | .846 | 10 | .051 |
| MPO.1 | .185 | 10 | .200^*^ | .846 | 10 | .051 |
| CXCL8.1 | .139 | 10 | .012 | .807 | 10 | .011 |
| CXCL8.2 | .139 | 10 | .012 | .807 | 10 | .021 |
| FN1.1 | .250 | 10 | .077 | .867 | 10 | .092 |
| FN1.2 | .250 | 10 | .077 | .867 | 10 | .092 |

Supplement 3. Clinical information of the RNA-seq group

| Patient | 1 | 2 | 3 | 4 | 5 | 6 |
| --- | --- | --- | --- | --- | --- | --- |
| Age | 65 | 63 | 70 | 65 | 66 | 68 |
| Eye | Left | Right | Left | Left | Right | Right |
| Gender | Man | Female | Female | Man | Man | Female |
| Lens | C1 | C1 | C1 | C4 | C4 | C4 |
| eye | / | / | / | Both | Both | Both |
| systemic comorbidities | Na | Na | Na | Na | Na | Na |
| medication use | Na | Na | Na | Na | Na | Na |
| Smoking | Na | Na | Na | Na | Na | Na |
| BMI | 22.3 | 20.1 | 23.2 | 22.9 | 21.2 | 22.6 |

| Control | 1 | 2 | 3 | 4 | 5 | 6 | 7 | 8 | 9 | 10 |
| --- | --- | --- | --- | --- | --- | --- | --- | --- | --- | --- |
| Age | 65 | 63 | 70 | 65 | 66 | 68 | 62 | 67 | 63 | 69 |
| Gender | Man | Female | Female | Man | Man | Female | Femal | Femal | Man | Man |
| R/L  (nucleus) | NC2/NC2 | NC2/NC3 | NC3/NC2 | NC2/NC2 | NC3/NC3 | NC2/NC2 | NC2/NC3 | NC2/NC2 | NC3/NC3 | NC3/NC2 |
|  |  |  |  |  |  |  |  |  |  |  |
| systemic comorbidities | Na | Na | Na | Na | Na | Na | Na | Na | Na | Na |
| medication use | Na | Na | Na | Na | Na | Na | Na | Na | Na | Na |
| Smoking | Na | Na | Na | Yes | Na | Na | Na | Na | Yes | Na |
| BMI | 21.8 | 22.4 | 20.5 | 23.4 | 21.1 | 22.3 | 21.9 | 20.7 | 20.9 | 21.5 |

Supplement4. Clinical information of the control group

Supplement5. Clinical information of the cataract group

| Cataract | 1 | 2 | 3 | 4 | 5 | 6 | 7 | 8 | 9 | 10 |
| --- | --- | --- | --- | --- | --- | --- | --- | --- | --- | --- |
| Age | 65 | 63 | 70 | 65 | 66 | 68 | 70 | 66 | 64 | 65 |
| Gender | Man | Female | Female | Man | Man | Female | Man | Man | Femal | Femal |
| eye | Both | Both | Both | Both | Both | Both | Both | Left | Both | Both |
| R/L  (cortex) | C3/C4 | C4/C4 | C3/C4 | C4/C2 | C3/C5 | C2/C4 | C4/C4 | C3/C4 | C4/C3 | C2/C5 |
| systemic comorbidities | Na | Na | Na | Na | Na | Na | Na | Na | Na | Na |
| medication use | Na | Na | Na | Na | Na | Na | Na | Na | Na | Na |
| R/L  (nucleus) | NC2/NC2 | NC2/NC3 | NC3/NC3 | NC2/NC2 | NC3/NC2 | NC2/NC3 | NC3/NC2 | NC2/NC2 | NC3/NC3 | NC2/NC3 |
| smoking | YES | Na | Na | Na | Yes | Na | Na | Yes | Na | Na |
| BMI | 22.8 | 21.2 | 21.9 | 22.9 | 23.1 | 20.6 | 20.3 | 22.3 | 21.5 | 21.7 |

**Supplement 6**
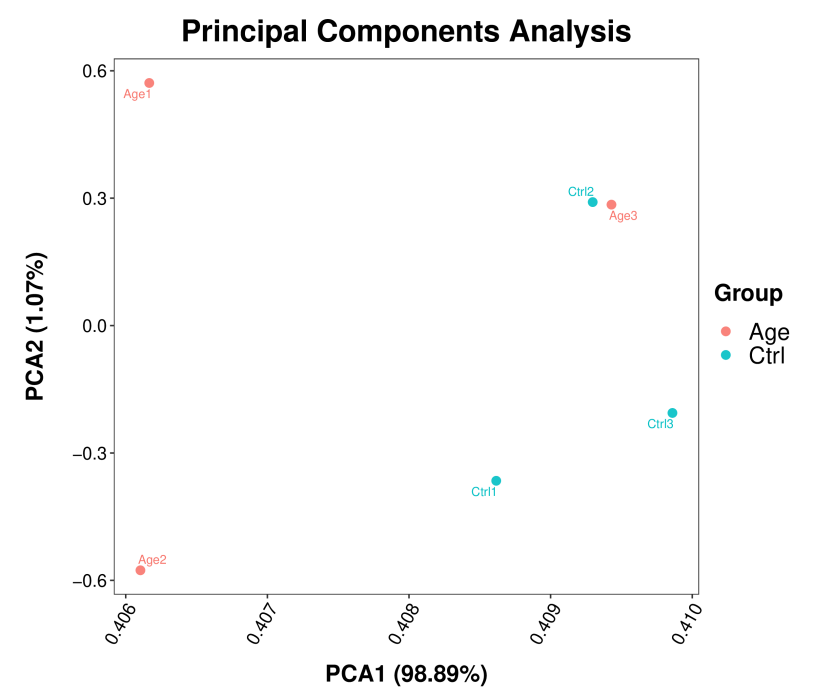


Red represents the ARCC group, blue represents the control group, and each dot represents one sample. The first two principal components, PCA1 and PCA2, explained 98.89% and 1.07% of the total variance, respectively.Samples from different groups show distinct clustering patterns along PCa1, indicating clear separation between these conditions.
